# Effector Functions of CD4+ T Cells at the Site of Local Autoimmune Inflammation—Lessons From Rheumatoid Arthritis

**DOI:** 10.3389/fimmu.2019.00353

**Published:** 2019-03-12

**Authors:** Karine Chemin, Christina Gerstner, Vivianne Malmström

**Affiliations:** Division of Rheumatology, Department of Medicine, Karolinska Institute, Karolinska University Hospital Solna, Stockholm, Sweden

**Keywords:** rheumatoid arthritis, CD4+ T cells, effector function, T-cell subsets, helper, cytotoxic, tissue-resident

## Abstract

Infiltration of memory CD4+ T cells in synovial joints of Rheumatoid Arthritis (RA) patients has been reported since decades. Moreover, several genome wide association studies (GWAS) pinpointing a key genetic association between the HLA-DR locus and RA have led to the generally agreed hypothesis that CD4+ T cells are directly implicated in the disease. Still, RA is a heterogeneous disease and much effort has been made to understand its different facets. T cell differentiation is driven by mechanisms including antigen stimulation, co-stimulatory signals and cytokine milieu, all of which are abundant in the rheumatic joint, implying that any T cells migrating into the joint may be further affected locally. In parallel to the characterization and classification of T-cell subsets, the contribution of different effector T cells to RA has been investigated in numerous studies though sometimes with contradictory results. In particular, the frequency of Th1 and Th17 cells has been assessed in the synovial joints with various results that could, at least partly, be explained by the stage of the disease. For regulatory T cells, it is largely accepted that they accumulate in RA synovial fluid and that the equilibrium between regulatory T cells and effector cells is a key factor in controlling inflammation processes involved in RA. Recent phenotypic studies describe the possible implication of a novel subset of peripheral T helper cells (Tph) important for T-B cell cross talk and plasma cell differentiation in the RA joint of ACPA+ (autoantibodies against citrullinated proteins) RA patients. Finally, cytotoxic CD4+ T cells, historically described as increased in the peripheral blood of RA patients have attracted new attention in the last years. In view of the recently identified peripheral T-cell subsets, we will integrate immunological data as well as information on genetic variants and therapeutic strategy outcomes into our current understanding of the width of effector T cells. We will also integrate tissue-resident memory T cell aspects, and discuss similarities and differences with inflammatory conditions in skin (psoriasis) and mucosal organs (Crohn's disease).

## Introduction

Rheumatoid Arthritis (RA) is a chronic inflammatory disease targeting peripheral joints leading to bone erosion, impairment of mobility, and decreased quality of life. It is affecting 0.5–1% of the population worldwide and is more prevalent in women than in men (1). The pathogenesis of RA is mainly localized in the synovial joint where immune cells composed of T cells, B cells, macrophages, and dendritic cells infiltrate the synovium. Moreover, fibroblast-like synoviocytes present in the sublining layer of the synovium proliferate and contribute to cartilage damage (2). Memory CD4+ T cells are enriched in affected joints of RA patients (3) and highly expanded CD4+ T cell clones are found in synovial tissue of early disease (4) suggesting that T cell expansion could be due to local antigen-induced proliferation. The efficiency of co-stimulation blockade targeting CD80/CD86-CD28 interaction further illustrates the importance of T cells in the pathogenesis of RA (5).

A central function of CD4+ T cells in RA has also been deducted from genetic studies. An early report by Stastny (6) identified an association between RA and HLA-DRB1 that was further confirmed by genome-wide association studies (GWAS) (7). This association led to the “shared epitope hypothesis” whereby a five-amino acid sequence found in certain HLA-DRB1 alleles was associated with increased susceptibility to RA (8). In about 2/3 of RA patients, serum antibodies to citrullinated protein antigens (ACPAs) are present and these are associated with the HLA-DRB1 risk alleles (1). Altogether, these findings have led to the hypothesis that citrullinated peptides might be preferentially presented by HLA-DRB1 risk alleles (9). Such peptide presentation has indeed been demonstrated both functionally (10) and by peptide-HLA crystal structure determination (11). Several citrullinated candidate peptides can be presented by HLA-DRB1^*^04:01 and other shared epitope alleles such as ^*^04:04 and ^*^10:01 (10, 12, 13) and the search for immunodominant T cell epitopes is still an important area of investigation in the field of RA. The relevance of antigen specificities has already been discussed elsewhere (13) and will not be detailed in this review but instead will be discussed in the context of effector T cell functions.

**Figure 1:**
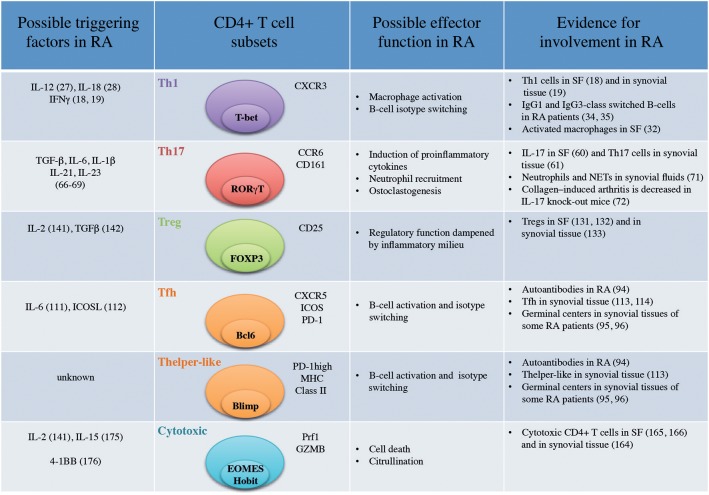
Important CD4+ T-cell subsets in Rheumatoid Arthritis (SF, Synovial Fluid; NETs, Neutrophil Extracellular Traps).

Infiltration of CD4+ T cells at the site of inflammation is a characteristic feature of several autoimmune syndromes. In the scope of this review, we present and discuss up-to-date understanding of effector functions of CD4+ T cells (Figure 1) present in the joint of RA patients. Examples of CD4+ T cell effector functions from other chronic inflammatory conditions (psoriasis and Crohn's disease) are selected to contrast and discuss our current knowledge in the field of RA. In particular, many common therapeutic strategies have been evaluated in RA, psoriasis and Crohn's disease with different outcomes that shed light on the different pathways implicated in the pathogenesis of these inflammatory disorders. Due to lack of space, this review will be mainly dedicated to findings in human inflammatory conditions.

## Th1 Cells and Associated Effector Functions

In 1986, Mosmann and Coffman proposed that mouse CD4+ helper T (Th) cells could be subdivided in Th1 or Th2 subsets based on their differential capacity to secrete IFNγ, IL-2, and TNF or IL-4, and IL-5, respectively (14). Subsequently, several reports identified human T cell clones separating into Th1 and Th2 categories (15). Th1 CD4+ T cells are crucial in the defense against intracellular pathogens such as mycobacteria (16) whereas Th2 CD4+ T cells mediate the immune defense against parasites such as helminths (17).

### Th1 Cells in Circulation and at Site of Inflammation

CD4+ T cells prone to secrete IFNγ (18, 19) were identified in synovial fluids from RA patients while IL-4 production (18) and IL-4+ T cell clones (19) were not increased in synovial fluid compared to peripheral blood. RA was subsequently defined as a Th1-driven disease while Th2 immunity was proposed to have a therapeutic potential in RA (20). CXCR3 was identified as a surface marker for Th1 cells (21) and T-bet as a master transcription factor (22). CXCR3 binds the two IFNγ-induced chemokines CXCL9 and CXCL10 (23). CXCR3 expression on CD4+ T cells (24) as well as CXCL9 and CXCL10 are enriched in synovial fluids (25). Although the vast majority of CD4+ T cells present in synovial joints are of memory phenotype (CD45RO+) (3) and hence antigen-experienced, our insight into their antigen specificity is scarce. Non-specific CD4+ T cells infiltrating the inflamed joint are likely to bias the analysis of the phenotype of relevant CD4+ T cells.

In that context, *ex vivo* peptide-HLA-DR-tetramer analysis provides a more relevant picture of antigen-specific i.e., citrulline-reactive T cells. Hereby, around 40% of citrulline-reactive CD4+ T cells were found to be CXCR3+ in the blood of RA patients (26) pointing again toward a Th1 signature of autoreactive T cells in RA. Presence of IL-12, IL-18, IFNγ, drivers of Th1 differentiation has also been reported in the synovial tissues of RA patients but not in osteoarthritis patients (Figure 1) (27, 28). However, there is still a lack of information concerning the phenotype of antigen-specific CD4+ T cells at the site of inflammation. Finally, immunodominant T cells epitopes have yet to be discovered in RA that will facilitate the more common use of peptide-HLA-DR-tetramer.

### Downstream Effects of Th1 Activity

Th1 cells classically induce macrophage activation (29) characterized in the context of the synovial joint by an increased capacity to produce pro-inflammatory cytokines such as TNF (30). Long-lived resident macrophages are present in synovial tissues from healthy donors (31) while inflammatory macrophages are mainly derived from blood monocytes in active RA (32). The interplay between Th1 cells and these two different subsets of macrophages in the context of the synovial joint is unknown. It will be particularly important to understand if Th1 cells can modify the properties of resident macrophages which could then contribute to perpetuation of the disease (33). Th1 cells have been proposed to influence class switching toward IgG1 and IgG3 in humans (20). In RA, polyclonal antibodies against type II collagen are predominantly of IgG1 and IgG3 subclasses (34) and autoantibodies against citrullinated fibrin are mainly IgG1 (35) suggesting previous interaction with IFNγ-producing cells. Nevertheless, Ig class switching is probably influenced by a multitude of other factors during the course of inflammation and should not be oversimplified by a link to a specific CD4+ T-cell subset. T helper cells also provide help to CD8+ T cells as demonstrated in the context of cancer immunology (36). Despite a reported presence of CD8+ T cells in synovial joints (37), the influence of CD4+ T cells on their activation is currently unknown.

### Th1 Targeted Therapy

Evidences of pathogenic function of Th1 cells in RA were contradicted by the lack of efficiency of therapeutic strategy targeting IFNγ (Fontolizumab) initiated in a phase II clinical trial in active RA. This clinical trial was terminated because the first phase did not reach the goals of primary endpoint (38). In the same line, in IFNγ receptor knock-out mice, collagen-induced arthritis was accelerated (39). In this particular mouse model, it has been proposed that IFNγ suppresses inflammation through inhibition of Th17 responses (40). It is however currently unknown if this hypothesis holds true in a human setting. It should be mentioned that biologic therapies targeting TNF, a Th1 cytokine are successful treatments in RA (41). Hence, Th1 cells could act on at least two opposing levels by directly contributing to tissue damage through TNF production or by suppressing Th17 responses.

Since Th1 cells were one of the first T helper cell subsets described, their contribution to the pathogenesis of autoimmune diseases has been investigated in numerous studies. This is also the case both for psoriasis (42, 43) and Crohn's disease (44) that were both initially suggested to be Th1-driven diseases. IFNγ-producing cells were indeed identified at the site of inflammation in these two diseases (42, 45). However, in a phase II clinical trial, Fontolizumab did not induce a robust beneficial clinical effect in Crohn's disease (46). Similarly, in a small study, therapeutic targeting of IFNγ with a humanized anti-IFNγ (HuZAF) showed no significant efficacy in psoriasis patients (47). In these three diseases, despite the clear presence of Th1 cells at the site of inflammation, therapeutic targeting of IFNγ did not lead to beneficial results. IFNγ might be important in the very early phases of the disease through, for instance, the induction of TNF in macrophages (48). It has also been shown that IFNγ induces the expression of vascular cellular adhesion molecule-1 (VCAM-1) on endothelial cells (49) which facilitates lymphocyte migration to the tissue. Finally, through reciprocal regulation, Th1 cells might also suppress the generation of pathogenic T cells such as Th17 cells that contribute to tissue damage.

## Th17 Cells and Associated Effector Functions

The Th1/Th2 hypothesis was revisited in 1995, when a third T-cell subset named Th17 cells based on the production of the newly identified cytokine IL-17 (50, 51) was discovered. The IL-17 family comprises 6 members with IL-17A (historically referred as IL-17) and IL-17F being the most closely related, in addition to IL-17B, IL-17C, IL-17D, and IL-17E (52). Th17 cells were initially described as co-expressing the chemokine receptors CCR6 and CCR4 (53) and expressing the master transcription factor RORγT (Figure 1) (54). In addition, CD161 was recently described as a marker of all IL-17 producing cells (55). In epithelial, endothelial and fibroblastic cells, IL-17A stimulates the production of pro-inflammatory cytokines such as IL-6, IL-8, and GM-CSF (56) and promotes neutrophil recruitment (57). Th17 cells are particularly important in protective immunity against fungal and extracellular bacterial infections (*Staphylococcus aureus*) (58).

### Classic Th17 Responses and RA

Early on, production of IL-17 was demonstrated in synovial tissues (59) and synovial fluid (60) of RA patients but not in that of osteoarthritis patients. In addition, IL-17-producing CD4+ T cells from synovial tissues from RA patients could readily be identified (61). The reported frequency of Th17 cells in peripheral blood of RA patients varies according to different studies where either an increase (62, 63) or a status quo in their frequency (64, 65) has been observed. Moreover, only few citrulline-specific CD4+ T cells were CCR6+ positive in peripheral blood of RA when analyzed by *ex vivo* peptide-HLA-DR-tetramer analysis (26). Th17-inducing cytokines (IL-6 IL1-β, IL-21, TGF-β, and IL-23) (66–69) are present in the synovial joint (Figure 1). Further, synovial IL-17 from RA patients was shown to induce bone resorption (70). Finally, IL-17 contributes to neutrophil recruitment, a hallmark of RA synovial fluid (71). In IL-17-deficient mice, collagen-induced arthritis was decreased supporting the notion that Th17 cells play a pathogenic role in the development of the arthritis (72). It was therefore unexpected that therapeutic targeting of IL-17A (Secukinumab) or the IL-17 receptor (brodalumab) in phase II studies was less successful in RA than in other inflammatory conditions such as psoriasis (73, 74). It was recently proposed that Th17 cells might migrate to the synovium in CCP+ (anti-cyclic citrullinated peptide) early RA patients (75). Hence, in RA, IL-17-producing T cells might contribute during early stages of the disease or be more prominent in a subtype of RA patients.

### The Different Facets of IL-17- Anti vs. Pro-inflammatory Features

Another level of complexity arises from evidence that Th17 cells are implicated in different immune responses depending on co-expressed cytokines (76). Indeed, T cells co-expressing IL-17 and IL-10 are thought to be important in mucosal defense but not pathogenic as T cells co-expressing IL-17, IFNγ, or GM-CSF are (66). After anti-TNF treatment, Th17 cells were shown to acquire IL-10 production in RA (77) implying that Th17 cells could also be protective and participate in dampening inflammation in RA. While GM-CSF appears to be a critical component of Th17 pathogenicity in the experimental autoimmune encephalomyelitis (EAE) mouse model (78), it is associated with the Th1 axis in multiple sclerosis (79). Likewise, in synovial joints of RA patients, GM-CSF production is enriched in Th1 cells and not in Th17 cells (80). Several clinical trials targeting GM-CSF are ongoing in RA (81) and will shed light on the pathogenic function of GM-CSF in the context of RA. It also implies that additional markers for Th17 pathogenic subsets are needed to predict which patients are more likely to respond to such therapies.

### Different Responses to Anti-IL-17 Blockade in Inflammatory Diseases

While the importance of Th17 cells seems to vary according to the stage and subsets of RA, psoriasis vulgaris can currently be defined as an IL-17-mediated inflammatory skin disease (82). IL-17-secreting cells are found in psoriatic lesions and include CD4+ (83), CD8+ (84), and γδ T cells (85). Phase III trials with a human anti-IL-17A monoclonal antibody (Secukinumab) were successful in psoriasis with response rates of 72–82% at week 12 (86). Th17 cells are also present in the gut of patients with Crohn's disease (87) but IL-17 blockade with Secukinumab was not effective and adverse cases of fungal infections and worsening of the disease were observed (88). In this case, IL-10-producing Th17 cells having a regulatory function (89, 90) might have been targeted contributing to the exacerbation of the disease.

Although early studies suggested that Th17 cells are crucial in most of human inflammatory conditions, different responses to IL-17 blockade contradict this hypothesis. Th17 cells are present at the site of inflammation in RA, Crohn's disease and psoriasis but the difference in the response to anti-IL-17 therapies strongly suggests that their direct implications in the pathogenesis of these diseases differ and reflect different effector functions in tissues. In psoriasis, blocking of IL-17 will alleviate the direct effects of IL-17 on keratinocyte proliferation. In the gut, Th17 cells play an important role in mucosal host defense (58, 91), which is reflected by the secondary effects observed in Crohn's patients under anti-IL-17A therapies. Th17 cells also produce IL-22 which is involved in intestinal epithelial barrier integrity (92). In active RA, targeting IL-17 might not be sufficient to dampen the ongoing cytokine cascade and resorb migration of neutrophils already sequestered in the synovial joint. Targeting of IL-17 at earlier time points or in conjunction with other anti-cytokine blockade might be more effective. Indeed, the combination of IL-17 and TNF synergize to induce the expression of P and E-selectins on endothelial cells that induce an influx of neutrophils (93).

## T Cells Providing B Cell Help–Tfh and Tph

Local CD4+ T-cell help to B cells is likely to be a prominent driver of humoral immunity in RA patients seropositive for ACPA (anti-citrullinated protein antibodies) and/or RF (Rheumatoid Factor). About 60–70% of RA patients present with ACPAs and 50-80% of patients are seropositive for RF (94). Already in 1992, synovial T cells from RA patients were shown to induce B cell Ig production *in vitro* (3). Moreover, ectopic germinal centers are observed in synovial tissues of some RA patients (95, 96). In 2000, a subset of follicular CD4+ T cells (Tfh) expressing CXCR5 and specialized in stimulating antibody responses was described in germinal centers in secondary lymphoid organs (97). They typically express the master transcription factor Bcl-6 as well as IL-21, PD-1, and ICOS. IL-6 and ICOS triggering regulate their differentiation (98) (Figure 1).

### B Cell Helpers in Circulation

Circulating Tfh can be analyzed in peripheral blood where their characteristics slightly differ from the follicular ones with a lack of expression of Bcl-6 (98–100). Blood Tfh can be further subdivided into Th1, Th2, Th17 cell subsets with Tfh2 and Tfh17 being the only subsets capable of sustaining the B-cell Ig switch (101). Several studies have described an increased frequency of CXCR5+ICOS+CD4+ Tfh cells in peripheral blood of RA patients which correlates with serological anti-CCP titers and disease severity (102–105). This is accompanied by increased concentrations of IL-21 in the serum of RA patients when compared to healthy donors (102–106). Few reports have investigated the frequency and function of CXCR5+ follicular T cells in psoriasis and Crohn's disease probably due to the lower prevalence of humoral immunity in these patients as compared to RA patients (107, 108). One study reported an increase of Tfh17 CD4+ T cells in peripheral blood of psoriasis patients that correlated with disease activity (109). An increased frequency of Tfh1 and Tfh17 CD4+ T cells has been observed in peripheral blood of patients with Crohn's disease (110).

### B Cell Helpers in Tissue

IL-6 (111) and ICOS expression on CD4+ T cells (112), inducers of Tfh differentiation, have been reported in the rheumatic joint. In synovial tissues, few CD4+ T cells express CXCR5 (113, 114) which is surprising given the reported expression of CXCL13, the ligand for CXCR5, in synovial fluids and tissues of RA patients (115, 116). It has recently been proposed that another subset of memory T cells, the peripheral T helper cell (T_ph_) subset expressing MHC class II and high levels of the co-inhibitory receptor PD-1 provide B-cell help in the synovial joint (Figure 1) (113). These cells lack Bcl-6 but express other typical markers for B-cell help such as IL-21, CXCL13, ICOS, and MAF. It is currently unknown if this subset shares the same ontogeny as Tfh. This study supports earlier reports showing the importance of PD-L1 (program death ligand-1)/PD-1 interaction in RA. Indeed, most of C57BL/6-Pdcd1-/- mice develop arthritis (117) and CD4+ T cells were shown to express PD-1 in synovial joints of RA patients (118). CD4+PD-1+ infiltrating T cells have also been described in the context of breast cancer (119) where they display comparable features with T_ph_ such as ICOS and CXCL13 expression (120). Importantly, these cells also express IFNγ in both RA synovial fluid (113) and breast cancer (119) showing that these T cells have the capacity to convey multiple effector functions contradicting the original Th1/Th2 hypothesis. Recently, the occurrence of case reports of RA following PD-1 or PD-L1 blockade in the context of cancer therapies have also highlighted the role of this checkpoint regulation in the balance between cancer and autoimmunity (121). Cases of psoriasis have also been reported (122) suggesting the importance of PD-1 in the pathogenesis of this disease as well. Although this subset of pathogenic T cells has not been extensively studied in psoriasis, PD-1 expression on Th17 cells has been reported in psoriatic skin of patients (123). So far, the subset of pathogenic T_ph_ cells driving B-cell responses has only been described in the synovial joint of RA patients but is probably relevant to other antibody-positive autoimmune diseases.

## Regulatory T-Cell Subsets

The concept of regulatory T cells was revisited in 1995 when the group of Sakaguchi described a population of CD4+ CD25+ T cells capable of preventing the development of several autoimmune diseases in mice (124). Shortly after, the key function of the transcription factor FOXP3 (Forkhead box P3) in the development and function of regulatory T cells was demonstrated in mice (125) and humans (126). Regulatory T cells (Tregs) also express CTLA-4 (cytotoxic T lymphocyte-associated antigen-4) and other markers e.g., GITR (glucocorticoid-induced TNFR family related gene) and CD39 whose expression might vary depending on the context (127). In peripheral blood, Tregs can be divided into naïve and activated regulatory T cells based on the expression of CD45RA (128). Importantly, in humans, FOXP3 expression is not strictly restricted to regulatory T cells as it is transiently up-regulated also in effector T cells (129). Therefore, in humans, regulatory T cells cannot only be defined by the phenotypic expression of FOXP3 and CD25 but need to be supplemented by assessment of their *in vitro* suppressive capacity and/or the demethylation of the *FOXP3* TSDR (Treg cell-specific demethylated region) (130).

### Tregs at Site of Inflammation

In RA patients, FOXP3+CD25+CD4+ T cells accumulate in synovial fluid (131, 132) and in synovial tissue (Figure 1) (133). *In vitro* suppressive capacity and demethylation of the *FOXP3* TSDR showed that these regulatory T cells are functional (133, 134). However, effector T-cell proliferation and pro-inflammatory cytokines have been demonstrated to reduce FOXP3 regulatory T-cell function *in vitro* (134), which is likely to happen also *in situ*. Indeed, adding TNF during co-culture experiments was shown to inhibit regulatory T-cell functions (135, 136). Whether this effect is mediated through a direct action on effector T cells or regulatory T cells is still a matter of debate (137) since TNF can also induce conventional T-cell proliferation (138). The ontogeny of FOXP3+CD4+ T cells in synovial tissues is still unknown. FOXP3+ Tregs can originate from the thymus (thymus Tregs or tTregs) or be induced *in situ* from conventional T cells during infections or other inflammatory processes (induced Tregs or iTregs) (139).

Regulatory T cells from peripheral blood and inflamed joints of juvenile arthritis patients were shown to harbor a different T cell Receptor Vβ usage than conventional T cells suggesting that Tregs would be generated independently of conventional T cells (140). Whether this is also the case in RA is currently unknown but IL-2 (141) and TGFβ (142), important for induced regulatory T-cell generation are present in synovial fluids of RA.

In plaques of psoriasis patients, the frequency of FOXP3+ CD4+ T cells is increased when compared to healthy skins (143) where only few Tregs are found in the dermis and epidermis (143). FOXP3+CD4+ T cells present in the psoriatic lesions also co-expressed IL-17 (143, 144). Hence, as for RA, it has been suggested that the inflammatory milieu through for instance IL-6 (145) may affect regulatory T-cell function.

### Therapeutic Interventions Affecting Treg Function and/or Frequencies

Current therapies given in RA that alleviate inflammation are therefore likely to restore or increase regulatory T-cell function. In RA patients treated with anti-TNF therapy, an increased frequency of CD4+CD25 high T cells was observed in responders when compared to non-responders (146). Similarly, an expansion of CD4+CD25+FOXP3+ T cells has been observed after anti-IL-6R blockade and was accompanied by a decrease in Th17 frequency (147). Treg and Th17 cells have opposite functions but their differentiation both rely on the presence of TGFβ. In the absence of pro-inflammatory cytokines such as IL-6, Treg differentiation is enhanced whereas in the presence of IL-6, Th17 differentiation is promoted. Hence, targeting IL6R contributes to increase the ratio between Treg and Th17 cells in RA patients (147). Recently, a therapeutic strategy based on the use of low-dose of IL-2 has been developed to directly induce the expansion of Tregs *in vivo* in autoimmune patients (148). This concept relies on the fact that Treg development and expansion is dependent on low levels of IL2R signaling (149). Clinical trials investigating such treatments in RA are currently ongoing.

### IL-10 Producing Tr1 Cells

Another subset of T regulatory cells named T regulatory type 1 cells (Tr1) is defined by their suppressive function combined with their capacity to produce IL-10 (150). So far, no unique cell surface marker specific for Tr1 cells has been identified but the expression of several markers such as ICOS, PD-1, CD49b, TIM-3, and LAG3 is increased on this subset (151). Tr1 cells have been extensively studied in the context of intestinal mucosal immunity and the prevention of colitis (152, 153). The importance of IL-10 in intestinal immunity is also illustrated by the identification of mutations in *IL-10, IL-10RA*, and *IL-10RB* genes in children suffering from inflammatory bowel disease (IBD) (153). The frequency of Tr1 cells (defined by production of IL-10 and low production of IL-2 and IL-4) was found to be decreased in peripheral blood and synovial fluid of RA patients when compared to osteoarthritis patients and healthy donors (154). However, IL-10 does not only have an anti-suppressive effect but is also involved in B-cell activation and antibody production (155) and is secreted by follicular helper T cells (156). Clearly, IL-10 alone is not sufficient to define Tr1 cells and additional markers are needed to understand their possible function in synovial tissues. As of today, Tr1 cells have been clearly implicated in intestinal mucosal immunity but their contribution to the synovial joint homeostasis is less clear.

## Cytotoxic CD4+ T Cells

Although not part of the general text book, cytotoxic features of CD4+ T cells have been observed already more than 20 years ago but were initially described in T cell clones (157) raising the concern that their generation might be an artifact due to repeated *in vitro* stimulation. However, the presence of CD4+ T cells with cytotoxic activities (CD4+ CTLs) has been confirmed *ex vivo* in human diseases driven by a variety of viruses like CMV (158) or dengue (159) as reviewed in Juno et al. (160). In healthy individuals, the frequency of peripheral CD4+ CTLs is usually very low (161).

### Cytotoxic T Cells in RA

In peripheral blood of a subset of RA patients, several groups have reported an increased frequency of a population of CD4+CD28null cells expressing perforin, granzymes, and other cytotoxic features (162–164) (Figure 1). Although CD4+ CD28null T cells are not enriched in synovial fluid, the presence of perforin+CD4+ T cells has been repeatedly reported in synovial fluids and tissues (164–166). No unique marker is associated with CD4+CD28null T cells but they express proteins related to their cytotoxic functions which are more commonly found in CD8+ CTLs and Natural Killer (NK) cells including granzyme B, granzyme A, and perforin-1. NK cell activating receptors such as NKG2D are also found on CD4+CD28null cells (167). Further investigation of CD4+CD28null cells or an updated approach of studying such cells in RA is warranted in the light of recent characterization of CD4+ CTLs at the single cell level (168, 169). In particular, the recently described transcription factor Hobit was identified in CD4+ CTLs where its precise function remains to be determined (170). We recently demonstrated that the transcription factor EOMES, implicated in terminal T-cell differentiation and the transcription of perforin-1 (171), is increased in CD4+ T cells from synovial fluids of RA patients (166). Using single cell transcriptomics, expanded T cell clones present in the synovium of RA patients were also shown to express EOMES and granzyme B when compared to circulating expanded clones (172). Antigen-specificity of CD4+ CTLs in RA is still debated although an increase in their frequency is more prominent in CMV-seropositive patients suggesting a link between CMV infections and the generation of this T-cell subset in RA patients (173). Nevertheless, repeated antigen stimulation, a classical feature of chronic inflammation, seems to be a constant feature in CD4+ CTL generation (160). Although CD4+ CTLs were initially suggested to derive from Th1 cells, it has also been proposed that they represent an independent lineage with CRTAM (class I-restricted T cell-associated molecule) as a possible marker for precursors of CD4+ CTLs (174). IL-2 and IL-15 as well as 4-1BB triggering are thought to favor their generation (160). Importantly, IL-2 (141) and IL-15 (175) are present in synovial fluids of RA patients while soluble forms of 4-1BB and 4-1BB ligand are increased in peripheral blood of RA patients (176). The functional implications of CD4+ CTLs interactions with HLA class-II expressing cells in synovial joints such as macrophages, dendritic cells, neutrophils (177), chondrocytes (178), or endothelial cells remains largely unknown. They might directly contribute to joint damage as it has been shown that CD4+ CTLs can directly lyse EBV-infected B cells (179). Another possibility is that they participate in the hypercitrullination of NETs (Neutrophil Extracellular Traps) through a perforin-dependent mechanism (180). We indeed observed that the level of ACPAs correlated with the frequency of perforin+CD4+ T cells in synovial joint of RA patients (166). It was recently shown that CIA was attenuated in granzyme A-/- mice (181) that presented reduced osteoclastogenesis. The source of granzyme A was not identified in this study but we have shown that CD4+ T cells producing granzyme A are present in synovial joints of RA patients (166). Granzyme A also stimulates monocytes to produce IL-6, IL-8, and TNF (182) which can contribute to increased inflammation in the RA joint. The presence of CD4+ CTL at the site of inflammation has been reported in several autoimmune diseases (183–185). Cytotoxic CD4+ T cells expressing NKG2D were identified in the lamina propria of patients with Crohn's disease (186). Likewise, perforin+ CD4+ T cells have been observed in skin lesions from patients with psoriasis (187).

### Therapeutic Strategies Affecting Cytotoxic CD4+ T Cells

TNF has been shown to repress the expression of the *CD28* gene (188). In an early study, the expression of CD28 was indeed increased on CD4+ T cells in RA patients undergoing anti-TNF therapy but markers of cytotoxicity were not investigated (189). Direct approaches to target cytotoxic CD4+ T cells can be achieved by targeting specific molecules expressed on these cells. For instance, an antibody targeting NKG2D induced a reduction in disease activity in some Crohn's disease patients in a phase II clinical trial (190). Cytotoxic CD4+ T cells also express CX3CR1, the receptor for fractalkine, a chemokine expressed on synoviocytes and endothelial cells of synovial tissues from RA patients (191). A phase II clinical trial is currently investigating the effect of fractalkine blockade in RA patients refractory to TNF inhibitors or methotrexate therapy. Clearly, the results of these new therapeutic blockades will bring new insights into the contribution of cytotoxic CD4+ T cells to RA.

## Tissue-resident Memory T cells

Tissue-resident memory T cells (Trm) are memory T cells that remain in a given tissue during a long period of time. They are well-described in mucosal tissues where they contribute to the first line of adaptive defense after re-exposure to a specific pathogen. For instance, influenza-specific resident memory CD8+ T cells have been described in the lung (192). The transcriptional signature of Trm cells differs from circulating T cells and includes genes important for their migration and retention in a given tissue (193). While the markers defining CD4+ Trm T cells are likely to slightly differ depending on the tissue, receptors such as CD69, CD49a, PD-1, and CXCR6 are commonly expressed (193). Persistence of memory T cells in tissues is beneficial in the rapid intervention against pathogenic infections infections but is also proposed to participate in the maintenance of pathogenicity in autoimmune inflammatory conditions.

### Trm T Cells in Disease

Psoriasis is the best example of a clear implication of resident memory T cells in the pathogenesis and resurgence of the disease. Indeed, Th17 Trm cells are present in recurrent psoriatic skin lesions and persist in resolved skin even after effective treatment (194). These data highlight the implication of Trm T cells in the reappearance of psoriatic lesions in a site-specific manner (194). The presence of T cells in perivascular areas of healthy synovial joints has been reported but is largely inferior to the number of T cells observed in mucosal tissues at steady state (195). Persistence of inflammation in synovial joints is observed in RA patients even in clinical remission (196) and might be indicative of Trm involvement in RA as described in psoriasis. Recently, CD8+ T cells with features of Trm cells such as CD69, PD-1, and CD103 have been identified in synovial fluids of juvenile arthritis patients (197). A fraction of CD4+ T cells express PD-1 (118) and CD69 (198) in synovial fluid of RA patients but whether these cells are bonafide Trm cells is so far unknown. Importantly, the peripheral T helper cell subset recently described in synovial joints (113) also expresses PD-1 and CD69 suggesting at least some overlap with resident memory T-cell markers. In synovial fluids, T cell clones with identical TCR sequences persist over time indicative of retention mechanisms in the joint (199). However, clonal T-cell expansions have not been studied in the context of Trm markers. In particular, the maintenance of Trm T cells in synovial tissues during the course of the disease and during relapses has not been assessed in RA. This set of experiments would provide information about the nature of effector T-cell functions implicated in tissue damage as exemplified by the persistence of Th17 Trm cells in psoriasis.

Given the recent discovery of Trm T cells, no specific therapeutic strategy is currently targeting this population. The persistence of Trm T cells in the plaques of psoriasis patients show that they probably resist current therapies. Hence, future therapies targeting the maintenance of resident T cells in tissues represent an attractive perspective.

## Genetic Risk Variants and T-Cell Subsets

The study of genetic risk variants can allow a better understanding of the pathogenesis of the disease and the cell subsets involved and also helps to validate therapeutic targets (200). The first genetic contribution to RA is located in the HLA-DRB1 locus (8). Genome-wide association studies have also identified 100 additional loci associated to RA (Table 1 and Supplementary Table 1) (201–203) that are predicted to target immune pathways. This set of gene loci does not correspond to a unique T-cell subset signature. However, epigenetic chromatin modifications (trimethylation of histone H3 at lysine 4) of RA-associated risk alleles are enriched in primary CD4+ regulatory T cells (201) suggesting that the function of this subset might be implicated in RA. Psoriasis represents a clear example where part of the 35 genetic loci can be assigned to the IL-23/Th17 pathway (204). Some of the genetic variants shared between psoriasis and Crohn's disease correspond to the IL-23 pathway and the T-helper 17 cell lineage (Table 1) based on gene ontology biological process analysis [Enrichr (205)]. Shared genetic variants between RA and Crohn's disease highlight a positive regulation of IFNγ secretion that might reflect part of the Th1 component of these diseases (Table 1). Many of the genetic variants associated with RA are not common to psoriasis or Crohn's disease emphasizing the importance of distinct mechanisms in the pathogenesis of the disease (Supplementary Table 1). For instance, genetic variants in the *IL-10* and *IL-10R* loci are only found associated with Crohn's disease, which is striking given the importance of IL-10 regulatory function in the intestinal barrier as well as in inducing IgA class switch (206). Similarly, a risk locus encompassing PADI4 (peptidylarginine deiminase type 4) is found only in RA (Supplementary Table 1). PADI4 controls citrullination processes that are highly relevant in RA where anti-citrullinated peptides antibodies (ACPAs) are commonly found. Still, a clear correlation between genetic risk variants in RA and a specific T-cell subset is lacking. Nevertheless, this can be explained by several factors. First, although some of the locus variants directly have an effect on the expression of the assigned gene (201) (eQTL (expression quantitative trait loci effect)), in most cases the functional consequences of the genetic variants have not been elucidated. We recently demonstrated that the *PTPN22* risk allele (rs2476601) favors the development of EOMES+ CD4+ T cells with cytotoxic features in RA (166). This finding and the fact that *EOMES* risk variants are associated with RA (Supplementary Table 1) suggest that cytotoxic T cells probably contribute to the disease. Second, RA is a complex disease that might encompass several sub-phenotypes with distinct stages and genetic signatures that are not uncovered in current GWAS. Finally, our understanding of genetic variants is evolving together with our knowledge on T-cell differentiation mechanisms and will be revisited in the light of emerging data on new T-cell subsets.

**Table 1:** Shared genetic variants associated to Rheumatoid Arthritis (RA), Psoriasis and Crohn's disease referenced at Immunobase corresponding to GO biological process (enrichr), *p* = adjusted *p*-value.

	**Psoriasis**	**RA**	**Crohn's disease**	**GO biological process**
Shared among the three diseases	*REL, TAGAP, TNFRSF9 TYK2, UBE2L3*	*REL, TAGAP, TNFRSF9 TYK2, UBE2L3*	*REL, TAGAP, TNFRSF9 TYK2, UBE2L3*	Interleukin-23-mediated signaling pathway (*p* = 0.04802)
				Negative regulation of interferon-beta production (*p* = 0.04802)
				Interleukin-27-mediated signaling pathway (*p* = 0.04802)
Shared between Psoriasis and RA	*ELMO1, ETS1 IRF4, TNFAIP3*	*ELMO1, ETS1 IRF4, TNFAIP3*		Regulation of toll-like receptor 3 signaling pathway (*p* = 0.01682)
				T-helper 17 cell lineage commitment (*p* = 0.01682)
				T-helper cell lineage commitment (*p* = 0.01682)
Shared between RA and Crohns' disease		*IFNGR2, IKZF3, IL2 IL6ST, IRF8, PTPN2 PTPN22, RASGRP1*	*IFNGR2, IKZF3, IL2 IL6ST, IRF8, PTPN2 PTPN22, RASGRP1*	regulation of tyrosine phosphorylation of STAT protein (*p* = 3.079e-7)
				positive regulation of interferon-gamma secretion (*p* = 0.0003406)
				interleukin-21-mediated signaling pathway (*p* = 0.0003774)
		*SPRED2, STAT4, YDJC*	*SPRED2, STAT4, YDJC*	
		*CD40, IL2RA, IL21*	*CD40, IL2RA, IL21*	
		*CXCR5, BACH2*	*CXCR5, BACH2*	
Shared between Psoriasis and Crohns' disease	*ERAP1, HLA-C, IL12B*		*ERAP1, HLA-C, IL12B*	Interleukin-23-mediated signaling pathway (*p* = 8.300e-7)
	*IL23R, NOS2, SOCS1*		*IL23R, NOS2, SOCS1*	
	*STAT3, STAT5A, STAT5B TNIP1, ZMIZ1*		*STAT3, STAT5A, STAT5B TNIP1, ZMIZ1*	Cellular response to interleukin-7 (*p* = 3.344e-8) Regulation of T-helper 17 cell lineage (*p* = 0.00007020)

## Conclusions and Future Directions

Rheumatoid Arthritis is a complex disease where several T-cell subsets have been proposed to be involved. During the last 10 years, new therapeutic trials as well as extended GWAS have provided new data to reinvestigate the contribution of T-cell subsets in RA. Based on the therapeutic intervention and the genetic data, RA cannot be classified as a Th17-driven disease such as for example psoriasis. Moreover, it has become clear that in human inflammatory contexts, CD4+ T cells harbor multiple effector function profiles that do not follow the classical dogma of T helper classification. IFNγ and IL-17 are present in synovial joints of RA patients but their blockade does not necessarily improve disease suggesting that their effector function is not rate-limiting for the downstream processes. These cytokines initiate a cascade of proinflammatory cytokines that may no longer be reversed by blocking IFNγ or IL-17 alone. Earlier targeting of these cytokines or combined therapeutic targeting of more downstream cytokines such as GM-CSF and TNF might be more effective. Th1 cells might also have already differentiated into cytotoxic CD4+ T cells capable of inducing cytotoxic damage and a cascade of proinflammatory cytokines. In that context, granzyme A represents a good candidate target since it induces osteoclastogenesis (181) as well as proinflammatory cytokines (182). In ACPA+ RA patients, recent identification of pathogenic T_ph_ cells driving B-cell responses show that, in addition to IL-10, these cells also produce IFNγ and perforin-1 (113). How these multiple effector functions are integrated during T cell/APC interactions is currently not known. Still, the elevated expression of PD-1 on this subset confirms that this co-inhibitory signaling pathway is important in RA and represents a possible target. The emergence of the concept of resident memory T cells capable of perpetuating the disease represent a breakthrough in the understanding of the mechanisms behind disease chronicity and might also favor the development of new therapeutics. Based on the comparison between these three inflammatory conditions, it is clear that some pathogenic pathways are common to these diseases while some others are very distinct and are probably a reflection of different tissue-mediated immunity components. These data should also encourage us to stratify RA patients in subgroups who might be more likely to respond to certain therapies based on the stage of the disease as well as the genetic variants associated. Moreover, a more common use of single cell technologies will allow the dissection of functional properties of rare CD4+ T cells present in inflammatory tissues. However, caution should be taken when analyzing T-cell subsets present in inflammatory tissues since bystander T cells can bias our view of pathogenic T cells. The presence of specific T cell types in inflammatory tissues does not imply that they are necessarily involved in the pathogenesis of the disease. Hence, the analysis of antigen-specific T cells might give a more accurate picture of important effector T-cell functions in the aforementioned inflammatory conditions. Finally, instead of targeting a distinct T-cell subset or effector function, an alternative approach would be to perform antigen-specific targeting and hence to target pathogenic T cells irrespective of their phenotype. We hope that, with this review, we provide a better understanding of current knowledge of CD4+ T-cell functions in RA and highlight the possible ways to identify pathogenic T cells that could be therapeutically targeted.

## Author Contributions

KC, CG, and VM discussed the content of the review. KC wrote the manuscript. CG and VM edited the manuscript.

### Conflict of Interest Statement

The authors declare that the research was conducted in the absence of any commercial or financial relationships that could be construed as a potential conflict of interest.
